# Transactions of the Odontological Society of Great Britain

**Published:** 1879-10

**Authors:** 


### ARTICLE III.

### *Transactions of the Odontological Society of Great Britain.*

ORDINARY MONTHLY MEETING, June 9th, 1879.

The President announced that Mr. Howarth had presented to the Museum an aluminium and rubber denture, which had been worn for fifteen years. The plate had not been struck up, but had been made by pouring melted aluminum into the cast; it had then been trimmed and vulcanized. Notwithstanding the number of years it had been in use it showed no signs of deterioration.

Mr. Storer Bennet showed a contrivance for twisting malplaced teeth, which he had found very useful. Being very simple, he had no doubt that something similar must have been adoped by others, though he had not been able to find any mention of such an apparatus either in the Society's Transactions or elsewhere. It consisted of a vulcanite plate, in which the rubber was thick behind the tooth to be moved; but cut away immediately behind the part to be drawn in. Through the thick rubber a tunnel was cut, extending from immediately behind the outstanding edge of the offending tooth to the lingual surface of the plate, and deeply countersunk at its posterior extremity. This tunnel was for the reception of a hook made of half-round gold wire; the front of the hook was adjusted to catch on the projecting edge of the tooth, whilst a small "jump ring" was soldered to the other end. The back of the tunnel being countersunk, the ring was protected from irritating the tongue. Two short slits were cut in the free edge of the plate opposite the posterior extremity of the tunnel, and their anterior extremities joined by a shallow grove on the palatine surface of the plate. A small elastic rubber band was drawn into the grooves and connected with the ring at the back of the hook. No harm could be done to the palate by the elastic band, as it lay in the groove, and was flush with the surface of the plate. By this plan no cumbersome and unsightly apparatus was visible in the front of the mouth. Although the traction was slight at any given time, yet, being constant, its actual moving power was very great. The teeth were moved with but little pain. It was easy of application, and by giving the patient a few extra bands, with instructions to change them every few days, many unnecessary visits were avoided.

Mr. Chas. Tomes exhibited a regulation plate, the invention of Mr. Palmer, of Cheltenham, which he said was one of the simplest and best he had yet met with. Mr. Palmer stated that he had succeeded in getting with this instrument an expansion of the whole arch varying from 1-16th to $\frac{1}{4}$ inch per month, with very few attendants.

The President then called attention to an apparatus made by Messrs. Winderling, of Milan, which had been sent for exhibition by Mr. C. J. Fox, and which had been designed to facilitate the packing of celluloid and vulcanite. It consisted of a light iron frame; in the center of this was a small platform on which the flask rested, raised about four inches from the table. Beneath the platform were two burners for gas or spirit,—one for heating the flask to 212° F., the other to heat the celluloid to 280° F. A roll of celluloid was placed in a cylinder attached to the face of the flask, and when sufficiently liquefied was forced into it by a piston worked by a screw with lever handles. The advantages claimed for the apparatus were:—First, that the plaster not being heated beyond 212° F. remained very hard, so that metal models were not required; secondly, the case being flasked in one solid body of plaster and never opened, there was very little chance of raising the bite or of displacing any teeth, bands, etc. To obviate the difficulty of removing pieces from the solid block of plaster small brass plates were inserted while flasking, in such a manner that the plaster easily separated into pieces on removal from the flask. There being no steam pressure, there was no danger from this source.

Mr. Oakley Coles said that, from his experience of the results obtained from other machines which had been designed for the same purpose, he could not help thinking that Mr. Winderling had introduced into this several unnecessary complications. He knew of several already in the market,—for instance, the machine invented by Mr. Gartrell, of Penzance, and that introduced recently by the Dental Manufacturing Company,—which would complete the process in considerably less than two hours. About twelve years ago a very similar apparatus had been brought before the profession by Mr. Payne, of Great Russel Street, but the results obtained by it were not so satisfactory as those obtained by the ordinary method. He failed to see how raising the bite could be avoided by this process more than in any other; this was due to the teeth sinking in the plaster, and this would be quite as liable to occur in the apparatus before them as in others with which they were familiar. It was, no doubt, a very ingenious piece of mechanism, and the inventor deserved great credit for having brought it to such perfection; but it appeared to him too elaborate and complicated, and not likely to give any better results than the simpler processes with which they were already acquainted.

Mr. Hutchinson said he had had the advantage of seeing the apparatus worked by Mr. Winderling himself, and he thought it offered more advantages than Mr. Coles had given it credit for. Although complicated in appearance it was really simple enough in structure. In the first place the plaster was never exposed to wet heat, nor above 212° F., and consequently remained very hard, so that it was impossible to squeeze the teeth into the plaster: then the celluloid entered the flask by an aperture only one-sixth the diameter of the piston; and when full the surplus escaped through three small openings at the back, so that there was no undue pressure on the teeth. And lastly, the uniform temperature at which the celluloid was kept rendered it much less liable to shrinkage. Still further to obviate this tendency, Mr. Winderling advised that the plate should always be kept in cold water when out of the mouth. He asserts that celluloid will never warp so long as it is kept moist.

Mr. Coles asked how it was that Mr. Winderling had come to the conclusion that dry heat was better than moist for working celluloid : most experimenters had found steam or oil best to work with.

Mr. Weiss said that he considered the point on which Mr. Winderling appeared to lay so much stress, that of working the celluloid at a temperature not exceeding 212°, was itself a great mistake.

The great objection to the use of celluloid was its liability to scratch and fray in the mouth. He had found that this liability was greatly diminished by submitting the celluloid to as high a temperature as possible, short of destroying its composition. A plate which had been kept for half an hour at a temperature of 280° would be found to be much harder and denser in structure than one which had not been heated beyond 212°.

Mr. Charles Tomes said he could not quite understand how the difference between the diameter of the piston and that of the aperture through which the celluloid was forced could reduce the pressure on the model?

Mr. Hutchinson admitted that he had been led into a fallacy on that point, but the three vents to which he had called attention were quite sufficient to prevent the pressure from being excessive.

Mr. Turner remarked that celluloid often showed a tendency to warp after it had been in use for some time; was this tendency diminished by Mr. Winderling's process?

The President answered that Mr. Winterling laid great stress on the importance of always keeping the plate moist. He advised that it should be kept in a metal case on a damp sponge, and that care should be taken never to allow it to get dry.

Mr. Oakley Coles said that in a paper he had read before the society about ten years ago, he had pointed out that celluloid behaved in this respect very much like bone. Alternate moistening and drying favored warping; but if the plate was kept in one state or the other but slight alteration in shape would occur.

Mr. Hutchinson showed a mouth mirror illuminated by the electric light, which had been designed by Messrs. Coxeter of Grafton-street; it was intended for use in dull or foggy weather. It was similar in some respects to that which had been brought forward by M. Trouve, of Paris, but differed from this in other important particulars. The battery was a "constant" one, producing a direct primary current of great quantity and intensity. No strong acids were used, the power being generated by the action of platinum and peroxide of manganese. The light was obtained in the same manner as Trouve's, by the incandescence of a short piece of platinum wire, but a rheostat was fixed in the handle by which the passage of the current could be regulated, and to prevent the fusing of the platinum. It would give a steady light for from 20 to 30 minutes. Messrs. Coxeter had also arranged a means of keeping the mirror cool, by passing a current of water round the back of it. The apparatus could also be used for cauterizing.

Mr. Coleman said it might be in the recollection of some of the older members that a similar apparatus was exhibited before the Society some years ago. The illumination was more powerful than that which Messrs. Coxeter were now showing, for it enabled one to see through the alveolar process so that the outlines of the fangs of the teeth were distinctly visible. He had not been able to find any notice of it in the Transactions, and could not then call to mind when or by whom it had been shown.

Mr. Robert Hepburn said that the apparatus spoken of by Mr. Coleman, was the invention of Mr. Hart, of Edinburgh, a very eminent practical electrician. He remembered its being exhibited about ten years ago, and Mr.

Coleman had not exaggerated its capabilities. It was a far more brilliant light than the present.

Mr. Oakley Coles said that M. Trouve's apparatus was intended primarily for use with the laryngoscope, and the great objection to it was that the platinum got melted occasionally by the intensity of the current, and the drops of fused metal falling on the vocal cords burnt holes in them. He was glad to see that in Messrs. Coxeter's apparatus this was guarded against, by enclosing the platinum in a glass tube. He knew that of late several individuals had been experimenting with the view of making the electric light available for mouth illumination, and he had no doubt their efforts would ultimately be successful.

Mr. Hutchinson showed a supernumerary tooth which he had removed from the mouth of a patient 13 or 14 years of age; it occupied the place of a permanent lateral. On examining it he found that the root was much expanded and filled with pus, and on washing this out he found what appeared to first sight to be a second tooth inside of it near the apex. The idea struck him that the missing permanent lateral had got shut up inside the supernumerary tooth, though it was difficult to imagine how such a thing would occur. But, on examining the specimen under the microscope, he found that what appeared to be a second tooth was an abnormal dipping down of the superficial enamel. The tooth, although at first simple, was really composed of two denticles, each with a separate pulp cavity. Similar cases were mentioned in Mr. Tome's work (p. 227, 1st ed.,) but they were stated to be very rare. Mr. Hutchinson desired to express his indebtedness to the curator for the assistance he had given in preparing and identifying the specimen.

Mr. Oakley Coles then showed, for Mr. Hatfield, of Old Burlington street, a large mass of salivary calculus which had been removed from the mouth of a lady about 70 years of age. Before removal it formed one mass, which almost covered three incisors, a canine, and a small bicuspid stump. It was supposed to have been in course of formation for about two years. [TO BE CONTINUED.]

